# Activins and Inhibins in Cardiovascular Pathophysiology

**DOI:** 10.3390/biom14111462

**Published:** 2024-11-18

**Authors:** Wenyi Tang, Zhilin Gu, Jiuqi Guo, Mingzhi Lin, Hongqian Tao, Dalin Jia, Pengyu Jia

**Affiliations:** Department of Cardiology, The First Hospital of China Medical University, Shenyang 110001, China; wytang@cmu.edu.cn (W.T.); zlgu@cmu.edu.cn (Z.G.); jiuqiguo@cmu.edu.cn (J.G.); linmingzhi@cmu.edu.cn (M.L.); 2023120665@cmu.edu.cn (H.T.)

**Keywords:** activin, inhibin, TGFβ, cardiovascular disease

## Abstract

Activins and inhibins, members of the transforming growth factor β (TGFβ) superfamily, were initially recognized for their opposing effects on the secretion of follicle-stimulating hormone. Subsequent research has demonstrated their broader biological roles across various tissue types. Primarily, activins and inhibins function through the classical TGFβ SMAD signaling pathway, but studies suggest that they also act through other pathways, with their specific signaling being complex and context-dependent. Recent research has identified significant roles for activins and inhibins in the cardiovascular system. Their actions in other systems and their signaling pathways show strong correlations with the development and progression of cardiovascular diseases, indicating potential broader roles in the cardiovascular system. This review summarizes the progress in research on the biological functions and mechanisms of activins and inhibins and their signaling pathways in cardiovascular diseases, offering new insights for the prevention and treatment of cardiovascular diseases.

## 1. Introduction

Cardiovascular diseases are the current leading cause of morbidity and mortality worldwide, affecting over five billion people. Global aging also presents greater challenges in the field of cardiovascular health [1]. Given the severe burden of cardiovascular diseases, the continuous exploration of the mechanisms behind them and the therapeutic targets is crucial.

The transforming growth factor β (TGFβ) family plays a highly relevant role in the cardiovascular system. The TGFβ family, highly conserved in animals, regulates fundamental cellular activities, including at least 30 members, such as TGFβ, activins, inhibins, nodal, bone morphogenetic proteins (BMPs), growth differentiation factors (GDFs), and anti-Müllerian hormone (AMH) [2,3]. Many members of the TGFβ family are expressed in the heart and act on various cells within it. They are involved in heart development, injury repair, and functional disorders, mediating pathophysiological processes such as cardiac hypertrophy, fibrosis, contractility, metabolism, angiogenesis, and regeneration [4,5]. The diversity of the TGFβ ligands and the interaction between signaling pathways underpin their complex and significant roles in the cardiovascular system [5]. Studies have shown that the cardiovascular system’s responses to TGFβ ligands are complex and context-dependent, with even minor variations having systemic impacts [2,4,6]. As a result, only a few candidate drugs directly targeting the TGFβ signaling pathway have entered early clinical trials [7,8]. The precise modulation of the TGFβ superfamily’s signaling pathways is of great significance in medical research and application, necessitating more specific studies of the members of the TGFβ superfamily.

**Figure 1:**
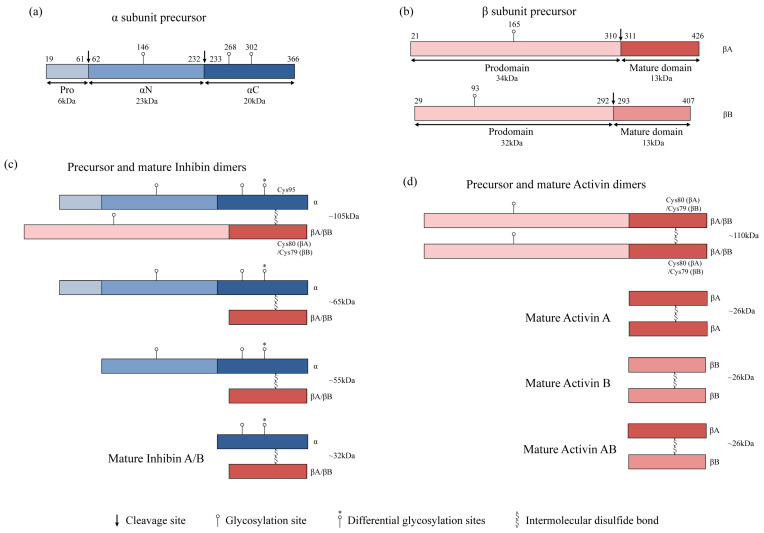
Subunit structures, precursors, and mature forms of inhibins and activins. (**a**,**b**) Diagrammatic representation of human inhibin α and β subunit precursor structure, respectively. Polyarginine cleavage sites are shown with arrows. (**a**) α subunits consist of a 43-amino-acid prodomain, a 171-amino-acid αN domain, and a 134-amino-acid αC domain. N-glycosylation sites are shown at positions 146, 268, and 302. Amino acid 302 shows differential glycosylation. (**b**) βA subunits consist of a 290-amino-acid prodomain and a 116-amino-acid mature domain, with the N-glycosylation site shown at position 165. βB subunits consist of a 264-amino-acid prodomain and a 115-amino-acid mature domain, with the N-glycosylation site shown at position 93. (**c**) Precursor and mature inhibin dimers. Inhibins are heterodimers of α and β subunits. (**d**) Precursor and mature activin dimers. Activins are homodimers of two β subunits. The cysteine residues at position 95 of the α subunit, 80 of the βA subunit, and 79 of the βB subunit contribute to intermolecular disulfide bonds. The molecular masses of the dimers are indicated in kilodaltons.

Activins and inhibins are members of the TGFβ subfamily, initially recognized for their opposing effects in reproduction and shared subunit pool; they are widely expressed in various tissue types, including the cardiovascular system [9,10]. The genes encoding the inhibin α subunit (INHA) and inhibin β subunit (INHB) produce monomeric α and β subunits, respectively. Mature inhibins (αβ) or activins (ββ) are heterodimeric or homodimeric proteins, linking certain subunits via disulfide bonds (Figure 1) [11,12]. Activins have been found to be involved in heart development [10,13,14], and both activins and inhibins have been reported to regulate angiogenesis in cancer [15,16,17]. Genetic studies suggest that mutations in activin and inhibin signaling may affect cardiovascular health [18,19,20]. It has been reported that the expression levels of activins and inhibins vary in various cardiovascular diseases [21,22,23,24]. Many studies have confirmed the close association between activin and inhibin signaling and the development of cardiovascular diseases [25,26,27,28,29]. In recent years, activins have been found to promote cardiac fibrosis and the progression of heart failure under pathological stimuli [23,29,30]. Moreover, many studies have reported contradictory biological effects of activins on other cardiovascular diseases [16,17,31,32]. Several gaps exist in research on the effects of inhibins on cardiovascular diseases. However, the signaling pathways of activins and inhibins found in other systems have been proven to exert an important effect in cardiovascular diseases [5,33,34]. This suggests that activins and inhibins may have more complex functions in the cardiovascular system. Further study of the roles and mechanisms of activins and inhibins in the cardiovascular system may be of great value. This article summarizes the progress of research on the roles and mechanisms of activins and inhibins and their signaling pathways in cardiovascular diseases.

## 2. Structure and Synthesis of Activins and Inhibins

Activins and inhibins, dimeric proteins formed by inhibin α and inhibin β subunits (also known as activin β subunits), were initially isolated from the gonads and named for their effects on the synthesis and secretion of follicle-stimulating hormone. They are widely expressed in human tissue and can be measured in the serum as endocrine and paracrine proteins [11]. Inhibins are heterodimeric proteins composed of inhibin α and β subunits, whereas activins are homodimeric proteins containing two β subunits. The subunit monomers are linked by disulfide bonds to form dimers and eventually form mature inhibin or activin glycoproteins [11,12]. The shared β subunits underlie the complex functional relationship between activins and inhibins [35]. To date, four β subunits, βA, βB, βC, and βE, have been identified, but biologically active dimer research mainly focuses on βA and βB subunits [35,36]. There are inhibin A (αβA) and inhibin B (αβB), and the dimerization of the two β subunits (βA or βB) forms activin A (βAβA), activin B (βBβB) and activin AB (βAβB) [11]. Research suggests that βC and βE subunits, forming homodimers like activin C (βCβC) and activin E (βEβE), are unlikely to signal through activin receptors. Instead, they might antagonize activin A signaling by forming heterodimers like βAβC or βAβE [37,38]. The study of activins and inhibins in the cardiovascular system mainly focuses on activin A and inhibin A.

Similar to members of the TGFβ family, activins and inhibins exist in precursor forms containing prodomains and mature domains, as well as in mature forms comprising only the mature domain (Figure 1). The characteristic structure of most TGFβ superfamily subunits includes seven conserved cysteine residues engaging in inter- and intramolecular disulfide bonds. Inhibin α has seven cysteines, while the inhibin β subunit has nine cysteines. The fourth cysteine can form a disulfide bond with the fourth cysteine of another subunit, stabilizing the homodimeric or heterodimeric forms [12,39]. The inactive precursor of the α subunit consists of three domains: a 43-amino-acid prodomain, a 171-amino-acid αN domain, and a 134-amino-acid αC domain, separated by two polyarginine cleavage sites (Arg-XX-Arg or RXXR). The cleavage of this precursor produces a mature form consisting of 134 amino acids with a molecular weight of 20 kDa. The precursor peptides of βA and βB subunits have 425 and 407 amino acids, respectively, with polyarginine cleavage sites dividing the N-terminal prodomains from the C-terminal mature domains. The cleavage of these precursors results in mature forms of 116 and 115 amino acids, respectively, each with a molecular weight of 13 kDa. The mature βA and βB subunits are approximately 64% identical but differ in 42 amino acids [12]. Mature α and β subunits both contain cysteine residues, which are crucial for the molecular stability and folding of the protein, necessary for intra- and intermolecular disulfide bonds. The C-termini of βA and βB subunits form inter-subunit disulfide bonds via Cys80 and Cys79, respectively, enabling the formation of activin dimers. Cys80 of the βA subunit (Cys79 of βB subunit) forms inter-subunit disulfide bonds with Cys95 of the α subunit, creating inhibin heterodimers. The polyarginine cleavage sites linking the prodomains and mature domains of precursors can be recognized and cleaved by proprotein convertases (such as furin), forming mature inhibins and activins [35]. Only the 26 kDa mature form of activin exhibits biological activity, while the 110 kDa form is inactive. Conversely, the mature 32 kDa inhibin and its 55 kDa and 65 kDa dimers are biologically active. This indicates that the cleavage of the α subunit precursor (pro-αN-αC) is not essential for the biological activity of inhibin A, whereas the processing of the βA subunit precursor is necessary for the biological response of activin A [40]. Reports suggest that the α subunit precursor monomer may also have some biological activity [41].

The assembly and secretion of TGFβ superfamily ligands depend on non-covalent interactions between their prodomains and mature domains. The conserved hydrophobic sequences in the prodomains of the α and β subunits of inhibins and activins influence these interactions, playing a role in controlling the dimerization and secretion of inhibins and activins. The hydrophobic residues at positions Leu30, Phe37, and Leu41 of the α subunit precursor peptide are crucial in the assembly of dimeric inhibins, with mutations at these sites affecting inhibins’ dimerization. Similarly, Ile62, Leu66, Phe329, and Pro341 of the βA subunit are implicated in the assembly of inhibin A and activin A [42]. The synthesis of inhibin A appears to depend on the expression of α subunit mRNA rather than the βA subunit that controls activin A synthesis [43,44]. However, this is inferred from protein transcription and expression levels and lacks direct experimental evidence. The assembly and secretion of inhibins also require the N-glycosylation of the α subunit. The human inhibin αC has two N-linked glycosylation sites at Asn268 and Asn302, responsible for the observed molecular mass heterogeneity between inhibin A and B. Asn268 is always glycosylated (31 kDa inhibin A or B), while Asn302 shows differential glycosylation (34 kDa inhibin A or B) (Figure 1) [41]. The mature α subunit is glycosylated, and the removal of this carbohydrate chain inhibits the formation of inhibins but does not alter activins [35,45]. Additionally, when mutations occur in the β subunit sequence, new glycosylation sites are incorporated to facilitate inhibin secretion, suggesting that the overall glycosylation levels can regulate the formation of activins and inhibins [46]. In studies of the reproductive system, epigenetic changes, such as DNA methylation and histone modifications, also play a role in controlling the expression of inhibin βA and βB subunit genes during ovulation and luteinization [47,48]. The current research focuses more on the concentration and modifications of the α subunit and its impact on inhibin synthesis. However, the way in which the β subunit choose to bind with an α subunit or another β subunit to form activin or inhibin dimers remains understudied, suggesting a potentially more complex regulatory mechanism.

## 3. Signaling Pathways of Activins and Inhibins

### 3.1. Activins and Inhibins Exert Biological Effects Through the Classical SMAD Pathway

As members of the TGFβ superfamily, the classical signaling pathways of activins and inhibins are also mediated through the TβR/SMAD pathway [5,49]. TGFβs signal through the TGFβ type I and type II receptors, both of which are transmembrane serine/threonine kinase receptors, along with coreceptors (type Ⅲ receptors) [49,50]. Seven type I receptors (activin-like kinase ALK1-7), five type II receptors (TβRII, ACTRIIA, ACTRIIB, BMPRII, AMHRII), and various coreceptors (such as endoglin and betaglycan receptors) have been identified [50]. Activins, inhibins, and nodal share type I receptors ALK2 (ACVR1), ALK4 (ACVR1B), and ALK7 (ACVR1C), with inhibins also potentially activating ALK1 (ACVRL1). Activins, inhibins, nodal, and BMPs share type II receptors ACVR2A and ACVR2B [51]. Upon binding to TGFβ family members, TGFβRII, as a high-affinity TGFβ receptor, recruits and phosphorylates the cytoplasmic domain of TGFβRI, activating SMAD proteins [9,49].

Activins have two β subunits and can symmetrically bind to two type II receptors and two type I receptors. The type II receptor binds with high affinity at the outer convex surface of each β subunit, and the type I receptor binds with low affinity at the interface of the two dimeric chains [9,35]. Activins, primarily activin A, directly bind to type II receptors (ACTRIIA or ACTRIIB) and recruit type I receptors (ALK4 and ALK7), predominantly ALK4. Activin B and activin AB can also recruit ALK7. Two type II receptors and two type I receptors form a heterotetramer, subsequently activating SMAD2/3 [11,52]. Some evidence also supports the notion that activins may activate SMAD1/5 [53]. The activated SMAD complex interacts with SMAD4, translocates to the nucleus, and influences the transcription of downstream target genes. Notably, different activin subtypes have varying affinities for type II receptors, with activin A having six times higher affinity for type II receptors than activin B [54]. Using βA and βB knockout mouse models, it was observed that activins A and B may have different functions in vivo, which may be related to differences in their affinities for receptors and antagonists, as well as their spatial and temporal expression patterns [55].

Although the activin signaling pathways have been relatively well studied, gaps exist in the study of inhibin signaling transduction. Inhibin has been observed to antagonize activin signaling in many systems, particularly in the pituitary–gonadal axis. The simplest mechanism involves inhibins binding with type II receptors through their β subunit, inhibiting activin activity via competitive binding [12,35,56]. The affinity of inhibins for type II receptors is about tenfold lower than that of activins, forming weaker inhibin–receptor complexes compared to activin complexes. This might be due to inhibins containing only one β subunit, hence recruiting only one type II receptor [9,57]. The low affinity contrasts with the observed strength of inhibin–activin antagonism, and some studies suggest that inhibins might compensate for this low affinity by interacting with the coreceptors through the alpha subunit [12,35,56]. Betaglycan and endoglin both are type III TGFβ coreceptors, which have structural similarities but differ in their cellular and tissue expression patterns [58,59]. Betaglycan (TβR III) appears to be primarily expressed in epithelial cells, while endoglin (ENG/CD105) is more highly expressed in proliferating endothelial cells [58,60]. They enhance the affinity of inhibins for type II activin receptors [9,12,35]. Betaglycan promotes the binding of inhibin and type II receptors and prevents the recruitment of type I receptors. Betaglycan sequesters inhibin type II receptors in the inactive complexes, blocks downstream activin signaling, and eventually functionally antagonizes the activin activity [35,56]. The knockdown or immunoneutralization of betaglycan eliminates inhibin’s antagonism function. Endoglin, on the other hand, forms complexes with endothelial type I receptor ALK1 and phosphorylates SMAD1/5, activating downstream signaling. Endoglin can negatively regulate SMAD2/3 and enhance SMAD1/5, so it is considered to regulate the signal balance of SMAD1/5 and SMAD2/3 (ALK1 and ALK5) [61,62,63]. Inhibin A has been shown to induce stable interactions between endoglin and ALK1, despite no direct binding of inhibin to ALK1 or endoglin being found [15,44]. Conversely, inhibin has been found to weaken the interaction between endoglin and ALK4 [59,64]. Inhibins also may have biological effects by signaling through their own receptors. In in situ radioligand studies, inhibin-specific binding sites were found in granulosa cells and ovine pituitary cells [65]. However, to date, no inhibin-specific binding molecules have been identified that support an activin-receptor-independent inhibin effect.

Contrasting with the large number of members and complex functions of the TGFβ family, the intracellular signaling pathways of the TGFβ family are highly convergent, involving five type II receptors, seven type I receptors, and two major SMADs. The specificity and diversity of TGFβ signaling, and its regulation of a range of critical cellular processes in a context-dependent manner, highlight the importance of specific studies on the differential cellular expression, interaction, and regulatory mechanisms of TGFβ family proteins and receptors [39].

### 3.2. Activins and Inhibins Exert Biological Effects Through Non-Classical Pathways

Like many other TGFβ family members, activins and inhibins also can activate SMAD-independent pathways in various cell types [35]. Some signaling pathways can also modulate SMAD activity, such as changing SMADs’ stability through the phosphorylation of MAP-kinase sites in the SMAD linker region [66].

Activins can exert biological effects through SMAD-independent pathways mediated by MAPK family members. Activin significantly inhibits the growth of human breast cancer T47D cells through an intact p38 MAPK pathway. The selective inhibition of p38 MAPK completely reverses the growth-inhibitory effects of activin [67]. Activin A, in combination with hepatocyte growth factor (HGF), strongly enhances the expression of ngn3 and the endocrine differentiation of AR42J-B13 (a rat pancreatic cell line). The beneficial effect on differentiation is weakened by the overexpression of a dominant-negative mutant of TAK1 or MKK3 or by treatment with a p38 MAPK-specific inhibitor. In contrast, the overexpression of the inhibitory SMAD7 does not affect activin/HGF-induced differentiation, suggesting that activin/HGF induce non-SMAD signaling through the TAK1-MKK3-p38 MAPK pathway, leading to pancreatic islet cell differentiation [68]. Activin A activates ALK4, which subsequently promotes the proliferation and differentiation of cardiac fibroblasts, in part through the ERK1/2 and p38 MAPK pathways [29]. Activin-induced MEKK1 activation leads to the phosphorylation of c-Jun N-terminal kinase (JNK), c-Jun, and p38 MAPK, resulting in stress fiber formation and cell migration. This effect of activin was observed in the keratinocytes of wild-type mice but not in MEKK1-deficient mice [69]. In pituitary prolactin-producing cells, activin inhibits the transcription of the Pit-38 promoter, requiring an intact p38 MAPK pathway [70]. Erythroid gene expression and cytokine-mediated colony formation in K562 leukemia cells are influenced by activin-induced p38 MAPK activation [71]. These studies suggest that non-SMAD-mediated activin signaling can impact cellular migration and differentiation processes.

Additionally, activin A can activate Akt, inhibit GSK signaling, and stimulate cell proliferation, leading to ovarian tumor development [72]. Activin signaling can crosstalk with the Wnt signaling pathway. Mice lacking activin βA exhibit mandibular molar arrest at the bud stage. In utero treatment with an agonist of canonical Wnt signaling, or the inhibitor of DKK, a secreted Wnt antagonist, rescued mandibular molar tooth morphogenesis in INHBA −/− embryos [73]. INHBA may act as an oncogene by activating the Wnt/β-catenin pathway, stimulating breast cancer cell proliferation, migration, and invasion [74]. In osteoclast precursors, activin A activates IκB-α and induces the nuclear translocation of p-NFκB, inducing RANK expression, leading to osteoclast differentiation [75].

Although there is still a gap in the study of the non-SMAD signaling of inhibins, studies on the receptors and functions of inhibins suggest that inhibins are likely to exert their biological effects through non-SMAD pathways. Exploring inhibins’ non-SMAD pathways may bring new insights for the research and application of inhibins and activins.

## 4. Pathophysiological Roles of Activins, Inhibins, and Their Signaling Pathways in the Cardiovascular System

### 4.1. Pathophysiological Roles of Activins and Inhibins in the Cardiovascular System

The in vivo, in vitro, and ex vivo studies assessing the roles and underlying functional mechanisms of inhibins and activins in cardiovascular pathophysiological changes are summarized in Table 1.

#### 4.1.1. Cardiac Development

The developing heart and vascular system express activins or activin receptors [76,77]. Moore et al. found that activin βA was expressed during mouse endocardial cushion formation and participated in endothelial cell transformation [10]. Kattman et al. found that activin/nodal signaling affected cardiac differentiation in mouse and human pluripotent stem cells [13]. The combination of activin A and BMP-4 has been found to facilitate cardiomyogenesis in serum-free human embryonic stem cell cultures, with the optimization of exogenous and endogenous signaling determining the efficiency of cardiac differentiation [13,14,78,79].

#### 4.1.2. Angiogenesis

Angiogenesis, a multi-step process, is regulated by the balance of numerous pro- and anti-angiogenic factors in the vascular microenvironment [95]. Inhibin has been demonstrated in tumor research as a novel paracrine factor for angiogenesis and metastasis, enhancing vascular formation by inducing the expression of pro-angiogenic factors [15]. Horst et al. found that tumor cell-secreted inhibin induced angiogenesis through SMAD1/5 signaling in endothelial cells, with endothelial-specific TGFβ receptor complexes containing ALK1 and endoglin being key mediators of inhibin signaling. Knockdown with shRNA and antibody therapy targeting inhibin is an effective anti-angiogenic strategy, leading to reduced vascular permeability and an increased vessel size but fewer vessels, potentially normalizing the vasculature [44].

The impact of activins on angiogenesis remains controversial. Maeshima et al. found that activin A increased the expression of VEGF and VEGF receptors in bovine aortic endothelial cells, enhanced VEGF-induced tubulogenesis, and induced capillary formation in this model [80]. Ervolino De Oliveira et al. also found a similar pro-angiogenic role for activin A in oral squamous carcinoma [16]. Bashir et al. found that activin A induced VEGF expression and promoted tumor formation in breast cancer [81]. Wagner et al. found that activin A stimulated VEGF gene transcription in human hepatocellular carcinoma cells [82]. However, this contradicts other studies. According to Kaneda et al., activin A inhibited gastric cancer endothelial cell growth and tumor angiogenesis by regulating p21’s transcriptional activity through SMADs [17]. Panopoulou et al. found that activin A downregulated the expression of vascular endothelial growth factor receptor-2, suppressed anti-angiogenesis, and reduced the vascularity in neuroblastoma [83].

#### 4.1.3. Hypertension

Hypertension is one of the major risk factors for fatal complications in cardiovascular diseases [96]. Pre-eclampsia, a hypertensive disorder that occurs during pregnancy, poses significant risks to maternal and fetal health and remains a leading cause of morbidity and mortality in pregnant women worldwide, characterized by hypertension and organ dysfunction [97]. Muttukrishna et al. found that the circulating levels of inhibin A and activin A were significantly elevated in women with diagnosed pre-eclampsia compared to those with normal pregnancies [21]. In early-onset pre-eclampsia patients, the serum levels of inhibin A and activin A can be significantly elevated before 20 weeks. The levels of activins and inhibins show better sensitivity in predicting early-onset pre-eclampsia than in all pre-eclampsia cases [84]. Reddy et al. demonstrated that the plasma levels of activin A and inhibin A in women with pre-eclampsia before labor induction were higher than in normal women before labor induction [85]. Barrero et al. found that inhibin A was upregulated in all pregnancies, while activin A increased in mid- and late pregnancy; they attempted to use activin A and inhibin A as potential biomarkers for early-onset pre-eclampsia based on a receiver operating characteristic curve analysis [98]. Shahul et al. found that the postpartum activin A levels correlated with an increasing left ventricular mass index and increasing mean arterial pressure, suggesting that activin A may be a tool for the identification and monitoring of hypertension in pregnant patients at risk for late postpartum cardiac dysfunction [86]. Guignabert et al. also found a correlation between pulmonary arterial hypertension (PAH) and increased levels of activin A and activin B in the serum and lungs [87]. Ryanto et al. considered activin/inhibin signaling as a key mediator in the development of PAH [99].

#### 4.1.4. Atherosclerosis

Atherosclerosis is a leading cause of death in developed and developing countries, considered a chronic inflammatory process caused by the interaction of atherogenic lipoproteins with vascular wall cells, including endothelial and smooth muscle cells [100]. Kozaki and Ouchi’s examination of the in vivo expression of activin A suggests its presence in various atherosclerotic lesions, including human coronary arteries, indicating that the activin A/follistatin system may play a significant role in the development of atherosclerosis [101]. Engelse et al. found that activin A was expressed in atherosclerotic lesions and promoted a contractile, non-proliferative phenotype in smooth muscle cells, inducing the re-differentiation of neointimal smooth muscle cells, contributing to plaque stabilization [88]. The overexpression of activin in both human saphenous vein organ cultures and a mouse model reduced the migration of vascular smooth muscle cells to the intima [102]. According to Liu et al., activin may play a role in inhibiting the progression of atherosclerosis [89]. Considering that activins and inhibins and their signaling pathways can influence the pathophysiological changes in the vascular endothelium and smooth muscle cells, we speculate that activins and inhibins may also play a role in chronic venous diseases, but this requires additional experimental evidence.

#### 4.1.5. Cardiac Fibrosis

Cardiac fibrosis, a terminal characteristic of almost all types of heart disease, is closely associated with fibroblast activation and ECM deposition. ECM deposition increases the risk of arrhythmias and impaired cardiac function, ultimately progressing to heart failure [103]. Activin A is a key regulator of cardiac fibrosis [29,30]. Venteclef et al. found that activin A induced atrial myocardial fibrosis in a rat atrial organ culture model [90]. According to Xian et al., the activin/ALK4/SMAD2/3 signaling pathway is involved in regulating endothelial-to-mesenchymal transition [104]. Hu et al. found that activin A also activated ERK1/2 and p38-MAPK, stimulating cardiac fibroblast proliferation and differentiation [29]. Castillero et al. found that activin signaling induced fibrosis and inflammation in patients and animals with heart failure post-myocardial infarction, contributing to cardiac remodeling [30]. Wei et al. used Ramipril to modulate the expression of activin A/follistatin in a rat model of heart failure, reducing fibrosis and collagen accumulation in the left ventricle post-myocardial infarction, being beneficial for ventricular remodeling [91]. Inhibin subunits have been reported to play a role in tissue fibrosis in many other tissue types, like the liver and kidney [105,106,107,108]. SMAD1/5 signaling also has been shown to inhibit tissue fibrosis in various tissue types [109,110,111]. However, the role of inhibin in cardiac fibrosis requires further research.

#### 4.1.6. Myocardial Infarction

Acute myocardial infarction, a primary cause of death in coronary heart disease, can be further exacerbated by reperfusion treatment, characterized by the irreversible death of cardiac cells and a restricted blood supply [112]. Lin et al. found that the activin A levels increased in ST-elevation myocardial infarction (STEMI). The activin A levels can predict worse left ventricular remodeling and all-cause death in STEMI [22]. There are conflicting reports on the role of activin A in myocardial infarction and ischemia–reperfusion injury. Dogra et al., through studies on zebrafish heart regeneration, proposed that the activin β subunit and activin type 2 receptor ligands play contradictory roles in myocardial cell proliferation during development and repair. The overexpression of INHBA in cardiac cells activates ACVR2A, promoting SMAD3 activation, inhibiting SMAD2, and facilitating cardiac recovery and scar clearance post-injury. Conversely, the activation of another ligand gene, MSTNB, leads to the activation of ACVR2B, promoting SMAD2 activation, inhibiting SMAD3, and resulting in reduced myocardial cell proliferation and impaired scar clearance post-injury [92]. Oshima et al. found that activin A was induced by myocardial stress in the heart. In vivo and in vitro overexpression experiments showed that activin A protected ischemic cardiac cells from apoptosis, reducing the infarct size in hearts with ischemia–reperfusion injury [31]. Roh et al. found that activin A induced cardiac atrophy and reduced the levels of the SERCA2a protein, reducing the myocardial energy demand and protecting the ischemic myocardium [23]. However, the cardioprotective effects of activin A have been questioned. Chen et al.’s study found that myocardial ischemia–reperfusion stimulated the local production of activin A through the activation of TLR4 signaling, potentially damaging cardiac cells independently of the reactive oxygen increase [32]. Magga et al. found that the systemic blockade of ACVR2B ligands protected the acute infarct myocardium; alleviated acute ischemia–reperfusion injury; reduced the infarct size, cell apoptosis, and autophagy after acute infarction; and better preserved the left ventricular contractile function. The systemic blockade of ACVR2B ligands antagonized SMAD2 signaling and cell death in myocardial cells stressed by hypoxia [93]. Castillero et al. also found that activin A promoted fibrosis and inflammation in patients and animals with heart failure post-myocardial infarction, inducing cardiac remodeling [30].

#### 4.1.7. Cardiac Aging

Advanced age is a significant risk factor for various cardiovascular diseases and events [113]. Roh et al. found that systemic ACTRII activity increases during human aging, correlating with the severity of heart failure. In mice, increased circulating activin A leads to enhanced ACTRII signaling and impaired cardiac function, potentially inducing heart failure. Conversely, blocking ACTRII in various heart failure models induced by aging, sarcomere mutation, or stress overload can restore or preserve cardiac function. ACTRII activation induces a pathological phenotype in cardiomyocytes, triggering heart failure transcriptional profiles, causing cardiac atrophy, reducing the levels of the SERCA2a protein, and possibly decreasing the myocardial energy consumption. While its early activation may offer short-term myocardial protection, chronic activation in aging and heart failure is maladaptive, ultimately leading to a cardiac function decline [23]. Similar effects have been observed in skeletal muscle studies, where activin drove SMAD2/3, acting on transcription-regulating E3 ubiquitin ligases and mediating muscle atrophy. Inhibiting activin production or activity promotes an increase in muscle volume and mass [114,115]. These studies suggest that activin signaling may play a significant role in the pathophysiological changes in the heart during aging.

#### 4.1.8. Heart Failure

Heart failure is a major component of morbidity and mortality in cardiovascular diseases, with an increasing prevalence due to global population aging [23]. Yndestad et al. found that the serum activin A levels were elevated in patients with heart failure, and activin A was localized to cardiomyocytes [24]. Roh et al. found that, although the acute increase in activin A may protect the heart in the short term, the long-term activation of activin A signaling impairs cardiac function and may lead to heart failure [23,24]. MacDonnell et al.’s in vivo and in vitro experiments revealed that activin A directly impairs the contractile function of cardiomyocytes. The overexpression of activin A in mice leads to heart dysfunction, increases cardiac stress markers, and causes cardiac atrophy. In vitro experiments showed that activin A causes an increase in SMAD2/3 phosphorylation in cardiomyocytes, leading to impaired contractility, prolonged relaxation kinetics, and dose-dependent spontaneous beating [94]. Paddock and O’Meara proposed that targeting the activin type II receptor could help to treat heart failure. The downregulation of SMAD2/3 through ACTRII inhibitors has certain significance in improving pathological cardiac remodeling [116]. Blumensatt et al. found that activin A impairs insulin’s action in cardiomyocytes and may participate in cardiomyocyte dysfunction development in type 2 diabetes [117]. Wei et al. used Ramipril to modulate the expression of activin A/follistatin in a rat model of heart failure, reducing left ventricular remodeling [91]. These studies indicate that the targeting of activin might have significant clinical potential in the treatment of heart failure.

### 4.2. Pathophysiological Roles of Activin- and Inhibin-Related Receptors and Signaling Pathways in the Cardiovascular System

Although some roles and the corresponding mechanisms of activins and inhibins in the cardiovascular system have been elucidated (Table 1), many research gaps remain. Their signaling pathways, confirmed in other systems, show strong correlations with cardiovascular health and diseases, indicating their great potential in the cardiovascular system. The functions of activin- and inhibin-related receptors and their signaling pathways in the cardiovascular system are summarized in Table 2.

#### 4.2.1. Pathophysiological Roles of Activin- and Inhibin-Related Receptors in the Cardiovascular System

Activins and inhibins function through TGFβ family receptors, activating classical SMAD pathways or other non-classical pathways. ALK1, in conjunction with endoglin, regulates blood pressure and vascular homeostasis [5]. ALK1 deficiency or abnormality can lead to abnormal angiogenesis and impaired differentiation [118]. ALK1 was also found to participate in atherosclerotic cardiovascular disease progression [119]. Morine et al. also found that ALK1 was involved in cardiac pathological remodeling, with reduced ALK1 expression promoting cardiac fibrosis and left ventricular dysfunction in a heart failure mouse model [25]. The ALK4 pathway plays a significant role in cardiac fibrosis, with the partial inhibition of ALK4 reducing cardiac fibrosis and improving cardiac function [120]. The knockdown of ALK4 post-myocardial infarction in mice improves ischemia–reperfusion injury by inhibiting the TGFβ signaling pathway [26]. ALK4 is also involved in cardiac remodeling, with ALK4 deficiency attenuating pathological cardiac hypertrophy and the susceptibility to atrial fibrillation through the inactivation of SMAD2/3 [121]. ALK4 inhibition can balance abnormal intracellular levels of Ca^2+^ in diseased cardiac cells, alleviating cardiomyopathy [27]. ALK7 exhibits a protective role in the heart. Silencing ALK7 in diabetic rats alleviated cardiomyocyte apoptosis, cardiac fibrosis, and cardiac dysfunction [122]. ALK7 prevented pathological cardiac hypertrophy in mice by negatively regulating MEK-ERK1/2 signaling [123]. In cardiac electrophysiology, ALK7 helps to maintain the repolarizing K+ currents in ventricular myocytes, preventing ventricular arrhythmias [124]. Gong et al. found that ALK7 may have potential roles in vascular diseases by negatively regulating PPARγ expression, promoting vascular smooth muscle cell phenotype modulation [125].

ACTRII signaling is associated with cardiac aging and heart failure [23,126]. As previously mentioned, Roh et al. found that ACTRII signaling increases with aging, frailty, and the severity of heart failure, regulating SERCA2a to modulate the cardiomyocyte energy demand and maintaining cardiac function under short-term stress but impairing cardiac function and leading to heart failure with long-term activation [23]. Castillero et al. found that inhibiting ACTRII signaling increases Akt activation and reduces p38 activation, attenuating cardiac remodeling and preventing fibrosis post-experimental ischemic heart failure in mice [30]. Moreover, Li et al. found that the blockage of activin signaling improves cardiomyopathy caused by neuromuscular diseases and diabetes [27], and Joshi et al. found that ACTRIIA-Fc improves cardiopulmonary remodeling and pulmonary arterial hypertension in experimental left heart failure [127].

Endoglin, a TGFβ coreceptor, plays a significant role in the cardiovascular system [28]. Expressed in endothelial and hematopoietic cells in mammals, endoglin is crucial for normal vascular structure formation, necessary for extra-embryonic angiogenesis, and plays a key role in heart development. Genetic mutations in endoglin are associated with hereditary hemorrhagic telangiectasia type 1 [128]. Endoglin, along with Alk5, regulates epithelial–mesenchymal transition during cardiac valvular formation [129]. Its expression increases during angiogenesis, wound healing, and inflammatory processes [28]. Polymorphisms in endoglin (rs3739817 and rs10987759) are associated with cardiovascular risks, such as higher levels of low-density lipoprotein cholesterol and increased hemoglobin levels and heart rates [130]. Endoglin activates the SMAD pathway to regulate the PI3K-Akt, Wnt, TNF, and metabolic pathways; modulates the apoptosis, proliferation, and migration of vascular endothelial cells; and participates in plaque formation [131]. Endoglin interference can affect the course of coronary atherosclerosis [131]. Endoglin mediates TGFβ signaling for pro-fibrotic actions on cardiac fibroblasts and regulates ECM synthesis. Reducing endoglin activity limits TGFβ signaling, mitigating cardiac fibrosis and improving survival [132]. Endoglin deficiency promotes heart failure development [133,134].

Betaglycan, as a coreceptor of the TGFβ pathway, promotes ligand binding to TβRII, modulating specific TGFβ/SMAD signals [59]. Betaglycan is required during cardiovascular system development. Betaglycan mutations in mice lead to fatal proliferative defects during heart development [135]. In myocardial infarction, betaglycan promotes ischemia-induced cardiomyocyte apoptosis, enlarging the infarct size [136]. It also negatively regulates TGFβ signaling, protecting post-infarction cardiac fibroblasts from apoptosis [137]. Numerous studies indicate that betaglycan overexpression can inhibit collagen production and attenuate cardiac fibrosis, suggesting that the targeting of this pathway has significant potential in preventing and treating cardiac fibrosis and myocardial remodeling [138].

#### 4.2.2. Pathophysiological Roles of Activin- and Inhibin-Related Signaling Pathways in the Cardiovascular System

SMAD proteins, mediating the classical signal transduction pathway, play a crucial role in various cardiovascular diseases. SMAD proteins have been shown to influence heart development, cell proliferation, growth, and apoptosis. TGFβ family members activate SMADs, leading to processes like apoptosis, fibrosis, and anti-hypertrophy, associated with pathological cardiac remodeling progressing to heart failure [2,5,139]. SMAD1 contributes to the protection of myocardial cells [139]. The activation of SMAD2/3 and 4 promotes cardiac fibrosis [139], whereas SMAD1/5 show antifibrotic effects [111]. Under pathological stimuli, different members of the TGFβ superfamily activate SMADs in cardiac diseases, activating other transcription factors related to SMADs, regulating cardiac cell apoptosis and fibrosis, and impacting the progression of heart failure and angiogenesis in the vascular system.

The MAPK signaling cascade plays a crucial role in cardiovascular health and disease, potentially regulating the heart’s hypertrophic response to pressure overload, promoting cardiomyocyte growth, participating in ischemia–reperfusion injury, and having a controversial role in cardiac remodeling. The ERK1/2, JNK, and p38 pathways, mediating activin and inhibin’s non-classical signal transduction, are representative MAPK pathways in the heart [33].

Alterations in Akt signaling play a significant role in various cardiovascular pathological processes. An exercise-activated PI3K Iα-induced increase in Akt activity is beneficial for cardiovascular health [140,141]. Several studies have demonstrated that PI3K/Akt signaling can reduce myocardial ischemic injury [142]. However, chronic Akt activation in the heart can impair cardiac function, and PI3K p110α can repair this damage in an Akt-independent manner [143]. Short-term Akt activation increases angiogenesis, while chronic Akt activation can decrease angiogenesis. The initial pro-vasculogenic effect of Akt activation promotes cardiac adaptation to cardiac hypertrophy, but, in later stages, Akt activation may lead to cardiac hypertrophy and heart failure [143]. The PI3K/Akt-mTOR pathway also promotes cardiac fibrosis in myocardial infarction mice [142].

The Wnt signaling pathway influences metabolic changes, cardiovascular remodeling, atherosclerosis formation, hypertrophy, and fibrosis in cardiovascular diseases [34]. In atherosclerosis, Wnt signaling is a key pathway in its progression [34]. The classical Wnt/β-catenin signaling pathway plays a leading role in the Wnt regulation of cardiac fibrosis after myocardial infarction and is involved in infarction healing. After acute ischemic injury to the heart, Wnt1/βcatenin upregulates and activates epicardial and cardiac fibroblasts to promote cardiac repair [144]. Wnt5A is associated with right ventricular dysfunction in dilated cardiomyopathy, promoting cardiomyocyte growth and protein synthesis, leading to cardiac hypertrophy [34].

NF-κB plays a complex role in the heart. During acute hypoxia and reperfusion injury, NF-κB protects against hypoxia-induced mitochondrial defects, preserving cardiomyocyte survival. However, the prolonged activation of NF-κB negatively impacts cardiac function [145]. NF-κB is involved in cardiac hypertrophy, with the silencing of NF-κB in mice helping to inhibit cardiac hypertrophy and remodeling [145]. NF-κB activation is associated with inflammatory cardiovascular diseases, and the inhibition of its activation has been proposed as a therapeutic intervention for heart failure and other related diseases [146].

## 5. Conclusions and Discussion

Activins and inhibins, as subfamily members of the TGFβ superfamily, activate the TGFβ receptor system and are involved in pathophysiological changes in the cardiovascular system through the classical SMAD pathway and various non-classical pathways. Within the activin and inhibin family, studies related to cardiovascular system disorders are limited, mainly focusing on activin A and inhibin A. However, sufficient evidence indicates the complex roles of the activin/inhibin system in cardiovascular diseases, with some studies presenting contradictory results. This may be due to insufficient content and methods for research on activin and inhibin and also suggests the complexity of the regulation of activin and inhibin and their signaling pathways.

Activins and inhibins share the inhibin β subunit pool but exhibit distinctly different physiological actions; thus, the regulation of their subunit assembly deserves attention. It is currently believed that the expression concentration of subunits is a factor in the regulation of the synthesis of activin and inhibin. The synthesis of inhibin A seems to depend on the expression of α subunit mRNA, rather than the βA subunit that controls the synthesis of activin A. However, this has been inferred from protein transcription and expression levels and lacks direct experimental evidence. The expression of activins and inhibins exhibits cellular specificity in many systems, possibly due to the cell-specific expression of the subunits affecting assembly. Nonetheless, many gaps exist in the research on the upstream regulation of the transcription and translation of activins and inhibins. Additionally, post-translational modifications could also impact the assembly and secretion of subunits. Studies have reported that the glycosylation of subunits affects inhibin assembly and secretion. The effects of other post-translational modifications on the assembly and secretion of activins and inhibins require further investigation.

Research approaches to activins and inhibins also have limitations. The currently available commercial antibodies target subunits, and, as dimeric proteins, many studies lack discussion on the detection of complete proteins. Often, they only reflect the level of a specific subunit, not directly representing the amount of a particular inhibin or activin. Furthermore, due to the shared β subunit, activins and inhibins display complex interrelations in function, highlighting the necessity of research into these proteins. The concentration of inhibin subunits has been reported to potentially affect the synthesis of activins and inhibins. Therefore, interventions targeting a single protein or subunit might simultaneously affect the expression of both activins and inhibins. The limitation of this property is particularly evident when performing in vivo experiments, where it is difficult to delineate the specific function of a single protein.

Similar to other TGFβ family members, activins and inhibins have complex and extensive biological functions. Limited research already suggests their impacts on the development and progression of the cardiovascular system, as well as on various pathophysiological changes, such as cardiac hypertrophy, cardiac fibrosis, and angiogenesis. They play complex and crucial roles in the development of various cardiovascular diseases, such as myocardial infarction and heart failure. However, reports on their specific biological actions are often contradictory, such as their effects on angiogenesis and cardiomyocyte protection, which have been reported as both positive and negative. This may be due to the research limitations mentioned above or the complex interactions and signal crosstalk between activins and inhibins, other TGFβ members, and other signaling factors. The specific mechanisms of action require further investigation.

Activins and inhibins, as secreted proteins that are detectable in serum, could serve as new biomarkers for cardiovascular diseases, potentially applicable in conditions like cardiac aging and pre-eclampsia. However, the specific relationship between the serum levels of activins and inhibins and the disease severity remains unclear. According to the results derived from other systems, the serum levels of activin A increase with age, while inhibin B is found to decrease with age [147,148]. Considering the changes in the expression of related receptors in the heart with age [23], the changes in the levels of activins and inhibins with age may be related to age-related cardiovascular diseases and may affect the occurrence and development of these diseases. Activins and inhibins have the potential to serve as biomarkers for pathophysiological changes in cardiac aging. In reproductive biology, activins play a central role. Activin A shows no significant gender differences at certain stages [37,149]. During development, the serum concentrations of activin A are higher in girls than in boys, while inhibin B is higher in boys, which may be related to the gender differences in follicle-stimulating hormone levels [150]. The gender differences in the serum levels of activins and inhibins may lead to different impacts on the cardiovascular system. Like estrogen, the roles of activins and inhibins in the cardiovascular systems of men and women may be distinct [151]. However, most experiments in the field of cardiovascular research currently focus on collecting data from a single gender or do not distinguish by gender. Investigating the levels of activins and inhibins in males and females separately may further clarify their specific roles in the cardiovascular system. The dysregulation of activins has been found to be associated with various female reproductive and pregnancy-related diseases [152]. Inhibins fluctuate with the female menstrual cycle and are observed at lower levels under certain pathological conditions in women [153,154]. Some studies have provided evidence of the correlation between activins and inhibins and pregnancy hypertension, but further in-depth research is needed. Activin A and inhibin A exhibit significant changes in pregnant women, holding substantial potential in the diagnosis of pre-eclampsia [84].

The expression and distribution of activin signaling receptors in different tissue types and cells play a crucial role in their complex functional effects. In the classical pathway, the significant affinity difference in activins and inhibins for activin type II receptors poses a challenge regarding the mechanism by which inhibins antagonize activins through binding to type II receptors. It has been found that the coreceptors β-glycan and endoglin that inhibin can bind to have different distributions in different cells, suggesting that the biological effects of inhibin in different cells may be realized through this differential distribution. New possible coreceptors have also been reported, and the search for coreceptors is of great value for the study of the inhibin signaling pathway. There are also many unknowns regarding the generation of different polymers of the three types of TGFβ receptors and the regulation of their binding to activin and inhibin, especially considering that they can also bind to other TGFβ family members and endocrine proteins. The complex regulation and crosstalk among the downstream signaling pathways of TGFβ receptors also necessitate research into the mechanisms of action of activins and inhibins, and there is great difficulty in achieving the precise regulation of their actions.

The TGFβ family has numerous members with complex and extensive functions, and some have been found to play significant roles in the heart. However, the specific actions of many TGFβ members in the cardiovascular system lack detailed research. The pleiotropic nature of activins and inhibins indicates that their roles in different systems are interconnected, and changes in one system may have a cascading effect on other systems. There are complex interactions and pathway crosstalk between TGFβ family members, and the modulation of pathways related to the cardiovascular system may bring about other toxic responses; therefore, clinical applications of interventions targeting TGFβ in the cardiovascular system remain elusive. Further studies targeting the specific biological functions and mechanisms of action of TGFβ family members, including activins and inhibins in the cardiovascular system, are crucial in understanding the development, diagnosis, and treatment of cardiovascular diseases.

## Figures and Tables

**Table 1:** In vivo, in vitro, and ex vivo studies assessing the role of inhibins and activins in different cardiovascular pathophysiological changes.

Cardiovascular Pathophysiological Changes	Refs.	Experimental Approach	Observed Phenotypic Changes	Main Findings
Cardiac development	Roberts et al. (1994) [76]	mRNA expression in postimplantation rat embryos (8–20 days)	βA mRNA signal was observed in heart during embryogenesis	
	Feijen et al. (1994) [77]	mRNA expression in mouse embryos	βA mRNA was abundant in heart in 10.5- and 12.5-day embryos. βA and βB expression was observed in blood vessels	
	Moore et al. (1998) [10]	mRNA expression in mouse embryos	βA during the initial phase of ECT, βB at later stages	βA promotes the formation of mesenchymal cells in the endothelial cushions
		WT or βA mouse cardiomyocyte CM induced ECT in mouse endothelial cells	βA: cardiac ECM ↓	
	Kattman et al. (2011) [13]	Activin A and BMP4 induced mouse and human ESC and iPSC differentiation	Certain concentrations of activin and BMP4: Flk-1/KDR/PDGFR-α ↑, cardiac mesoderm and cardiomyocyte generation ↑	Optimization of activin/nodal and BMP signaling is required for efficient cardiac differentiation
	Sa et al. (2012) [78]	Activin A and BMP4 induced H7 and H9 ESC lines’ differentiation	6 ng/mL activin A + 30 ng/mL BMP4: KDR/PDGFR-α H7 ↑ on day 5; 10 ng/mL activin A + 60 ng/mL BMP4: KDR/PDGFR-α H9 ↑ on day 6	Certain protocols of activin A and BMP4 induce efficient cardiac differentiation in the H7 and H9 ESC lines
	Lian et al. (2012) [79]	Ebs, activin A, and BMP4 or Gsk3 inhibitor induced β-catenin± or ALK± human PSC differentiation	β-catenin in the initial stage of PSC differentiation: cardiomyocyte specification ↓; β-catenin or Wnt after Gsk3 inhibition: cardiomyocyte specification ↑ ALK5 or ALK2: cardiomyocyte specification ↓; Gsk3 inhibition or activin A and BMP4: SMAD1/5 and SMAD2 activated at comparable levels	Activin/nodal and BMP signaling are necessary for cardiogenesis induced via modulating regulatory elements of the Wnt pathway
	Kim et al. (2015) [14]	CHIR with or without activin A and BMP4 induced human ESC H1 and H9 cardiomyogenesis	CHIR + low level activin A during day 0–1: cardiomyogenic efficiency ↑; CHIR + high activin A: DE differentiation ↑, cardiomyogenesis ↓; CHIR + low BMP4: cardiomyogenesis↓	Activin/nodal and BMP signaling are necessary during the earliest stage of CHIR-induced cardiomyogenic differentiation. Activin A and BMP levels modulate cell type specification
Angiogenesis	Singh et al. (2018) [15]	Tissue microarray of OVCA tumor cores	More inhibin A expression led to more microvessels per mm^2^ in ovarian cancer	Inhibin induces endothelial cell response through SMAD1/5 activation mediated by ALK1 and endoglin and angiogenesis in vitro and in vivo in tumor metastasis and vascularization
		In vitro culture of shControl or shINHA ovarian epithelial carcinoma cell lines; HMEC-1, MEECs, and HUVECs ± inhibin A protein with or without anti-inhibin α	shINHA: endothelial cell tube formation ↓, endothelial cell SMAD1/5 activation ↓; Inhibin A: endothelial cell tube formation ↑, with anti-inhibin α endothelial tube formation ↓, SMAD1/5 activated and SMAD2/3 not activated, epithelial cancer cells SMAD1/5 not activated; Inhibin A with endoglin or ALK1 inhibition: tube formation and SMAD1/5 activation ↓	
		shControl or shINHA SKOV3 cells or inhibin A injected into mice	shINHA: blood vessel formation ↓, tumor angiogenesis ↓, metastatic ↓; Inhibin A: blood vessel formation ↑, tumor angiogenesis ↑, metastatic ↑	
	Horst et al. (2022) [44]	In vitro culture of normoxia or hypoxia ovarian epithelial carcinoma cell lines, HMEC-1s, COS7, WT, and endoglin −/− mouse embryonic endothelial cells with or without inhibin or anti-inhibin	Inhibin A: angiogenesis ↑, endothelial cell migration and permeability ↑, endothelial cell contractility ↑, VE–cadherin internalization↑, ALK1–endoglin cell surface complexes ↑, ALK4–endoglin complexes ↓ Anti-inhibin α: angiogenesis↓, endothelial cell migration and permeability ↓; Inhibin A with anti-ALK1 or anti-endoglin: endothelial cell permeability ↓; Inhibin A with endoglin−/−: VE–cadherin internalization not changed	Inhibin promotes hypoxia-induced angiogenesis and stimulates endothelial cell migration and vascular permeability through ALK1/endoglin/SMAD1/5 in ovarian cancer
		Hypoxia-induced shControl shINHA Hey cells or Hey CM without inhibin or anti-inhibin injected into mice	Inhibin A: angiogenesis ↑, endothelial cell migration and permeability ↑, tumor growth ↑; Anti-inhibin α: angiogenesis↓, endothelial cell migration and permeability ↓, tumor growth ↓; shINHA: angiogenesis ↓, tumor growth ↓, vascular leakiness ↓, vessel size ↑, numbers ↓, promotes normalized vasculature	
	Maeshima et al. (2004) [80]	Activin A with or without VEGF treatment of bovine aortic endothelial cells with or without activin A antagonist	Activin A: induced tubulogenesis and capillary formation, enhanced VEGF-induced tubulogenesis and capillary formation, VEGF and VEGF receptor expression ↑; Activin A antagonist: inhibit VEGF-induced tubulogenesis and capillary formation, VEGF and VEGFR expression ↓; ACVR Ⅱ mutation: inhibit activin A or VEGF-induced capillary formation	Activin A enhances VEGF-induced tubulogenesis and induces capillary formation in bovine aortic endothelial cells
	Ervolino et al. (2020) [16]	A cohort of 95 patients with OSCC	Activin A expression in blood vessels is associated with poor prognosis of OSCC	Activin A is a predictor of the prognosis of patients with OSCC and contributes to angiogenesis in an autocrine and paracrine manner
		Activin A, activin A antagonist, or shRNA ± INHBA OSCC cell line CM treatment of HUVECs, shRNA ± INHBA HUVECs	Activin A: tube formation ↑ and proliferation ↑ in HUVECs, induced SMAD2/3 phosphorylation and VEGFA expression; Activin A antagonist: tube formation↓ and proliferation ↓ in HUVECs; shRNA-INHBA: tube formation ↓, proliferation ↓ and migration ↑ in HUVECs	
	Bashir et al. (2015) [81]	Activin A treatment or overexpression in MCF-7 cells and HEK-293T	VEGF expression ↑; activity of VEGF promoter ↑	Activin A induces VEGF expression in breast cancer
	Wagner et al. (2004) [82]	Activin A treatment of human hepatoma cell lines	VEGF expression ↑, activity of VEGF promoter ↑, triggers SMAD2 nuclear accumulation and SMAD2/Sp1 complex formation; SMAD2 overexpression: VEGF promoter activity↑, VEGF expression ↑; SMAD2 mutation: SMAD2 nuclear accumulation ↓, VEGF promoter activity ↓, VEGF expression ↓	Activin A stimulates VEGF gene transcription through Sp1/SMAD2 interaction in human hepatocellular carcinoma cells
	Kaneda et al. (2011) [17]	Activin A treatment of HUVECs and gastric cancer cell line	Proliferation ↓, tube formation of HUVECs ↓, induced SMAD2 and p21 phosphorylation, increased the binding of SMAD2/3 and SMAD4 to the p21 promoter, growth with sh-p21 ↓	Activin A suppresses tumor growth and angiogenesis in gastric cancer
		TK3/INHBA cells inoculated into mice	Tumor growth and angiogenesis↓	
	Panopoulou et al. (2005) [83]	±Activin A WAC2, activin A treatment of BBCE	Proliferation ↓, formed smaller xenograft tumors with reduced vascularity, angiogenesis ↓; With SMAD2 or SMAD3 expression, BBCE proliferation ↓; with SMAD3 or SMAD4 inhibition, BBCE proliferation ↑	Activin A suppresses neuroblastoma xenograft tumor angiogenesis partly via the SMAD pathway
Hypertension	Muttukrishna et al. (1997) [21]	Blood samples from 20 women in hospital with established pre-eclampsia and from 20 control pregnant women attending antenatal clinics, who were matched for duration of gestation, parity, and maternal age	Serum inhibin A, pro alpha C, and total activin A ↑ in pre-eclampsia compared to control pregnancies; inhibin B not increased	
	Muttukrishna et al. (2000) [84]	Blood samples from 1651 healthy nulliparous women recruited in the community in a prospective, longitudinal study	Serum inhibin A and activin A increased prior to pre-eclampsia and before 20 weeks in early-onset pre-eclampsia. Predictive sensitivities were better for early-onset pre-eclampsia	
	Reddy et al. (2009) [85]	Plasma samples from 10 normal pregnant and 10 pre-eclamptic women pre-labor; plasma samples from 10 normal pregnant and 10 pre-eclamptic women undergoing elective Caesarean section	Activin A and inhibin A in women with pre-eclampsia before labor induction were higher than in normal women before labor induction. Activin A and inhibin A levels declined rapidly with placental delivery. Activin A rose during labor in pre-eclampsia compared to pre-labor, but inhibin did not increase	
	Shahul et al. (2018) [86]	Prospective study of 85 women’s antepartum activin A levels with cardiac dysfunction at 1 year postpartum	Postpartum activin A levels correlated with abnormal global longitudinal strain ↑, left ventricular mass index ↑, mean arterial pressures ↑, and E’ values ↓	Activin A may be a tool for identification and monitoring of hypertensive pregnant patients at risk of late postpartum cardiac dysfunction
	Guignabert et al. (2023) [87]	Serum samples from controls and patients with newly diagnosed PAH (*n* = 80) at baseline and 3 to 4 months after treatment initiation	Adjusted hazard ratios for transplant-free survival for baseline activin A were 0.14 (95% CI, 0.03–0.61; *p* = 0.009), and those for follow-up measures were 0.23 (95% CI, 0.07–0.78; *p* = 0.019). Prognostic values of activin A were confirmed in an independent external validation cohort.	Activin A is a prognostic biomarker for PAH
Atherosclerosis	Engelse et al. (1999) [88]	Human vascular tissue specimens at various stages of atherogenesis	Activin expression ↑ in neointimal SMCs from the early onset of atherogenesis	Activin induces redifferentiation of neointimal SMCs, induces the contractile, nonproliferative phenotype in vitro
		Activin A treatment of human iliac artery or aorta SMCs	SM α-actin expression ↑ in aortic SMCs, SM α-actin expression ↑ and SM22α expression↑ in iliac artery SMCs	
	Liu et al. (2022) [89]	LDLR−/− mice on a Western diet with hepatic activin A or GFP expression	Activin A expression ↓; Activin A overexpression: plasma total and LDL cholesterol ↓, inflammatory cells in aortae↓, proliferating hematopoietic stem cells in bone marrow ↓, reduced atherosclerotic lesion and necrotic core area in aortae ↓, liver steatosis ↓	Hepatic activin A expression reduces inflammation, hematopoietic stem cell expansion, liver steatosis, circulating cholesterol, and fat accumulation, which may contribute to the observed protection against atherosclerosis
Cardiac fibrosis	Hu et al. (2016) [29]	Activin A with or without AngⅡ treatment of adult rat left ventricular CFs with or without activin A, ERK1/2, or p38MAPK inhibition	Activin A: CF proliferation ↑, differentiation ↑, collagen I expression ↑, ERK1/2 and p38-MAPK pathway activation ↑; activin A inhibition, ALK4 inhibition and p38-MAPK inhibition: CF proliferation ↓, differentiation ↓, collagen I expression ↓, ERK1/2 and p38-MAPK pathway activation ↓; ERK1/2 inhibition: CF proliferation ↓, collagen I expression ↓	Activin A promotes CF proliferation and differentiation via ALK4, partly through the ERK1/2 and p38-MAPK pathways
	Castillero et al. (2020) [30]	Sham and MI mice with or without ACTRII ligand inhibiting treatment	ACTRII/TGFBR inhibition: cardiac fibrosis ↓, CTGF ↓, type I collagen ↓, fibronectin ↓, α-smooth muscle actin ↓, and MMP-12 ↓	ACTRII/TGFBR signaling inhibition prevents fibrosis after experimental MI
		Post-MI serum with ACTRII/TGFBRI-inhibiting treatments in normal human ventricular CFs	Connective tissue growth factor (CTGF) ↓, type I collagen ↓, fibronectin ↓, α-smooth muscle actin ↓	
	Venteclef et al. (2015) [90]	Samples of EAT and SAT from 39 patients undergoing coronary bypass surgery induced rat atria in organoculture conditions with activin A or activin A neutralizing antibody treatment	The EAT secretome induced global fibrosis and highly expressed activin A in EAT. Activin A: atrial fibrosis ↑; Activin A blocked: atrial fibrosis ↑	Activin A from EAT promotes myocardial fibrosis
	Wei et al. (2016) [91]	Sham and post-MI HF rat model with or without Ramipril group	MI with Ramipril: collagen I and III deposition ↓, activin A and ActRII in the non-infarcted area of the left ventricle ↓ follistatin ↑	Ramipril benefits left ventricular remodeling by reducing fibrosis in the left ventricles of rats with downregulation of activin A expression
MI	Lin et al. (2016) [22]	278 patients with STEMI followed for a maximum of 3 years	High activin A level was associated with triglyceride level ↑, LVEF ↓, and left ventricular end diastolic ventricular volume index ↓ 6 months later: activin A > 129 pg/mL was associated with LVEF ↓ and left ventricular end diastolic ventricular volume index ↑ 3 years later: activin A > 129 pg/mL was a predictor of all-cause death (*p* = 0.022) but not of HF (*p* = 0.767)	Activin A level > 129 pg/mL predicts worse left ventricular remodeling and all-cause death in STEMI
	Castillero et al. (2020) [30]	Sham and MI mice with or without ACTRII ligand-inhibiting treatment	ACTRII/TGFBR inhibition in MI: preserved cardiac function, BNP ↓, phosphorylation of Akt ↑, p38-MAPK ↓, SERCA2a ↑, unfolded protein response markers ↓, cardiac fibrosis ↓ and fibrosis markers ↓	ACTRII/TGFBR signaling promotes fibrosis and inflammation in patients and animals with HF post-MI, inducing cardiac remodeling
	Dogra et al. (2017) [92]	Cryoinjury-affected zebrafish with or without INHBAA or MSTNB mutation	INHBAA overexpression: activation of ACVR2A, SMAD3 activation ↑, SMAD2 ↓, cardiac recovery and scar clearance ↑; INHBAA LOF: unresolved scarring after cardiac injury	Activin β subunit can benefit myocardial redevelopment and repair
	Oshima et al. (2009) [31]	Activin A overexpression or activin A treatment of hypoxia/reoxygenation-induced NRVMs	Activin A: apoptosis ↓, Bcl-2 ↑; Activin A or ALK inhibitor: abolished cell-protective effect of activin A	Activin A protects myocytes from apoptosis
		Systemic overexpression of activin A in IR injury mice	Bcl-2 ↑, SMAD2 ↑, infarct size ↓	
	Roh et al. (2019) [23]	Sham or TAC with or without activin A overexpression or systemic ActRII inhibition in aged C57BL/6 mice and MHCF764L mice and CM-specific ActRIIB−/− mice	Activin A overexpression: p-SMAD3 ↑, SERCA2a protein ↓, cardiomyocyte function ↓, Ca^2+^ cycling impairments ↓; ActRII inhibition: survival ↑, SERCA2a protein ↑	Activin A induces cardiac atrophy and reduces SERCA2a protein, reducing myocardial energy demand and protecting ischemic myocardium
		Activin A-induced NRVMs with or without ActRII inhibition	Activin A: SERCA2a mRNA and protein↓; ActRII inhibition: SERCA2a protein↑	
	Chen et al. (2014) [32]	Sham or IR in WT and TLR4−/− mice	Myocardial activin A ↑ following 30 min of ischemia and 2 h of reperfusion in wild-type mice TLR4(−/−) mice: myocardial activin A not increased Activin A antagonist: MI ↓	Activin A damages cardiac cells in myocardial IR
		Normoxic or hypoxia A NVCM with activin A treatment or activin A antagonist	Activin A: cellular injury ↑ after 3 h of hypoxia and 2 h of re-oxygenation, cardiomyocyte mitochondrial membrane potential↓, no effect on reactive oxygen species production Activin A antagonist: cellular injury ↓	
	Magga et al. (2019) [93]	Systemic ACVR2B blockade in hypoxia/reoxygenation-induced mice	ACVR2B blockade: infarcted area ↓, p-SMAD2↓, apoptosis ↓, autophagy ↓, preserved LV systolic function ↑, induced physiological hypertrophy, optimized cardiac metabolism	Systemic blockade of ACVR2B ligands is protective against cardiac IR injury
		Activin A induced or ACVR2B blockade in hypoxia adult rat ventricular cardiomyocytes and neonatal cardiomyocytes transfected with SMAD2/3 reporter or SMAD1/5/8 reporter	Activin A: cell death ↑, SMAD2/3 ↑, SMAD1/5/8 not activated ACVR2B blockade: cell death ↓, SMAD2/3 ↓	
Cardiac aging	Roh et al. (2019) [23]	Proteomics dataset from Framingham Heart Study	Activins increase in human aging and HF	Age-increased activin/ActRII signaling triggers HF progress
		Sham or TAC with or without activin A overexpression or systemic ActRII inhibition in aged C57BL/6 mice and MHCF764L mice and CM-specific ActRIIB−/− mice	Circulating activin A and cardiac ActRII signaling increase in murine aging Activin A overexpression: cardiac function ↓, p-SMAD3 ↑, SERCA2a protein ↓, cardiomyocyte function ↓, Ca^2+^ cycling impairments ↓; ActRII inhibition: systolic function in murine age-related HF models ↑, SERCA2a protein ↑	
		Activin A-induced NRVMs with or without ActRII inhibition	Activin A: SERCA2a mRNA and protein ↓; ActRII inhibition: SERCA2a protein ↑	
HF	Wei et al. (2016) [91]	Sham and post-MI HF rat model with or without Ramipril group	MI with Ramipril: collagen I and III deposition ↓, activin A and ActRII in the non-infarcted area of the left ventricle ↓ follistatin ↑	Ramipril benefits left ventricular remodeling by reducing fibrosis in the left ventricles of rats with downregulation of activin A expression
	Roh et al. (2019) [23]	Proteomics dataset from Framingham Heart Study	Activins increase in HF	Long-term activin/ActRII signaling activation impairs cardiac function and may lead to HF
		Sham or TAC with or without activin A overexpression or systemic ActRII inhibition in aged C57BL/6 mice and MHCF764L mice and CM-specific ActRIIB−/− mice	Circulating activin A and cardiac ActRII signaling increase in LV pressure overload Activin A overexpression: cardiac function ↓, p-SMAD3 ↑, SERCA2a protein ↓, cardiomyocyte function ↓, Ca^2+^ cycling impairments ↓; ActRII inhibition: preserves and restores systolic function, pathologic gene expression profiles ↓, survival ↑, SERCA2a protein ↑; CM-specific ActRIIB−/−: cardiac dysfunction ↓	
		Activin A-induced NRVMs with or without ActRII inhibition	Activin A: SERCA2a mRNA and protein ↓; ActRII inhibition: SERCA2a protein ↑	
	Yndestad et al. (2004) [24]	During 1999 to 2000, white patients with stable HF for >4 months NYHA II through IV with no changes in medication during the last 3 months, compared with healthy control subjects	Serum levels of activin A ↑, INHBA in T cells ↑ in HF patients according to disease severity	Activin A is involved in the pathogenesis of HF
		Rat model of MI-induced HF	INHBA and Activin receptors ↑ after MI, localized activin A solely to cardiomyocytes	
		Activin A-activated NRVMs	SMAD2 ↑, infarction healing and myocardial remodeling mediators of gene expression ↑	
	MacDonnell et al. (2022) [94]	Activin A overexpression mice	Produced cardiac dysfunction, cardiac atrophy, cardiac stress markers ↑	Inflammatory cytokine-induced upregulation of activin A by CFs directly impairs cardiomyocyte contractility, which may contribute to cardiac dysfunction
		Activin A treatment of human iPSC–cardiomyocytes with or without anti-activin A antibody	Activin A: SMAD2/3 phosphorylation ↑, contractility ↓, prolonged relaxation kinetics, induced spontaneous beating, maladaptive diastolic calcium handling; Anti-Activin A: abrogated maladaptive calcium handling and CM contractile dysfunction	
		Inflammatory cytokine-induced primary human CFs	Strong upregulation of activin A	

ECT, endothelial cell transition; CM, conditioned media; ECM, extracellular matrix; ESC, embryonic stem cell; iPSC, induced pluripotent stem cell; PSC, pluripotent stem cell; OVCA, ovary cancer; HMEC-1, human dermal microvascular endothelial cell; MEEC, murine embryonic endothelial cell; HUVEC, human umbilical vein endothelial cell; VEGF, vascular endothelial growth factor; OSCC, oral squamous cell carcinoma; BBCE, bovine brain-derived capillary endothelial; SMC, smooth muscle cell; CF, cardiac fibroblast; MI, myocardial infarction; CTGF, connective tissue growth factor; MMP, matrix metalloproteinase; EAT, epicardial adipose tissue; SAT, subcutaneous adipose; STEMI, ST-elevation myocardial infarction; LVEF, left ventricular ejection fraction; NRVM, neonatal rat ventricular myocyte; TAC, transverse aortic constriction; IR, ischemia–reperfusion; HF, heart failure; ↑ increase; ↓ decrease.

**Table 2:** The roles of activin- and inhibin-related receptors in the cardiovascular system.

Receptor	Cardiovascular Phenotypic Changes	Function
ALK1	Vasculogenesis and angiogenesis	Regulate blood pressure and vascular homeostasis; ALK1 deficiency or abnormality leads to abnormal angiogenesis and impaired differentiation.
	Atherosclerosis	Contribute to atherosclerotic cardiovascular disease progression; polymorphisms in ALK1 increase cardiovascular risk factors.
	Cardiac fibrosis	Reduced ALK1 promotes cardiac fibrosis.
ALK4	Cardiac fibrosis	Promote cardiac fibrosis; reduced ALK4 expression suppresses cardiac fibrosis; regulates epithelial–mesenchymal transition.
	MI	Knockdown of ALK4 improves ischemia–reperfusion injury.
	Myocardial remodeling	Induces cardiomyopathy, inhibits endothelial cell proliferation and vascular remodeling; ALK4 deficiency attenuates pathological cardiac hypertrophy.
ALK7	Diabetic cardiomyopathies	Induces cardiomyocyte apoptosis; silencing ALK7 alleviates cardiomyocyte apoptosis, cardiac fibrosis, and cardiac dysfunction.
	Cardiac hypertrophy	Prevents pathological cardiac hypertrophy.
	Cardiac electrophysiology	Prevents ventricular arrhythmias.
		Promotes vascular smooth muscle cell phenotype modulation.
ACTRII	Cardiac aging and HF	Impairs cardiac function, promotes HF; inhibiting ACTRII attenuates cardiac remodeling and prevents fibrosis post-ischemic HF.
	Myocardial remodeling	Inhibiting ACTRII attenuates cardiac remodeling, prevents fibrosis, and improves cardiomyopathy.
Endoglin	Vasculogenesis and angiogenesis	Contributes to vascular structure formation and angiogenesis, regulates blood pressure and vascular homeostasis; endoglin mutations contribute to hereditary hemorrhagic telangiectasia type 1.
	Cardiac development	Regulates cardiac valvular formation.
	Atherosclerosis	Modulates endothelial cell activity, participates in plaque formation; endoglin interference affects atherosclerosis; polymorphisms in endoglin increase cardiovascular risk factors.
	Cardiac fibrosis	Promotes cardiac fibrosis and regulates ECM synthesis; inhibiting endothelial attenuates type I collagen expression and cardiac fibrosis.
	HF	Endoglin deficiency promotes HF development; regulates the expression of transient receptor potential channels in HF.
Betaglycan	Cardiac development	Mediates epithelial–mesenchymal transition and coronary artery vessel development; betaglycan mutations lead to fatal defects during heart development.
	MI	Promotes cardiomyocyte apoptosis and enlarges infarct size; protects post-infarction cardiac fibroblasts from apoptosis and engages in fibrosis.
	Cardiac fibrosis and myocardial remodeling	Inhibits collagen production and attenuates cardiac fibrosis.

MI, myocardial infarction; HF, heart failure; ECM, extracellular matrix.
